# Establishing a knowledge structure for yield prediction in cereal crops using unmanned aerial vehicles

**DOI:** 10.3389/fpls.2024.1401246

**Published:** 2024-08-09

**Authors:** Ghulam Mustafa, Yuhong Liu, Imran Haider Khan, Sarfraz Hussain, Yuhan Jiang, Jiayuan Liu, Saeed Arshad, Raheel Osman

**Affiliations:** ^1^ Key Laboratory of Integrated Regulation and Resource Development on Shallow Lakes, Ministry of Education, College of Environment, Hohai University, Nanjing, China; ^2^ College of Agriculture, Nanjing Agricultural University, Nanjing, China; ^3^ College of Physics and Optoelectronic Engineering, Shenzhen University, Shenzhen, China; ^4^ Department of Agronomy, Iowa State University, Ames, IA, United States

**Keywords:** yield prediction, unmanned aerial vehicles, VOSviewer, wheat, maize, rice, soybean heading 3, left

## Abstract

Recently, a rapid advancement in using unmanned aerial vehicles (UAVs) for yield prediction (YP) has led to many YP research findings. This study aims to visualize the intellectual background, research progress, knowledge structure, and main research frontiers of the entire YP domain for main cereal crops using VOSviewer and a comprehensive literature review. To develop visualization networks of UAVs related knowledge for YP of wheat, maize, rice, and soybean (WMRS) crops, the original research articles published between January 2001 and August 2023 were retrieved from the web of science core collection (WOSCC) database. Significant contributors have been observed to the growth of YP-related research, including the most active countries, prolific publications, productive writers and authors, the top contributing institutions, influential journals, papers, and keywords. Furthermore, the study observed the primary contributions of YP for WMRS crops using UAVs at the micro, meso, and macro levels and the degree of collaboration and information sources for YP. Moreover, the policy assistance from the People’s Republic of China, the United States of America, Germany, and Australia considerably advances the knowledge of UAVs connected to YP of WMRS crops, revealed under investigation of grants and collaborating nations. Lastly, the findings of WMRS crops for YP are presented regarding the data type, algorithms, results, and study location. The remote sensing community can significantly benefit from this study by being able to discriminate between the most critical sub-domains of the YP literature for WMRS crops utilizing UAVs and to recommend new research frontiers for concentrating on the essential directions for subsequent studies.

## 1 Introduction

Wheat, maize, and rice were responsible for 30% of the world’s crop production by the year 2020. Furthermore, when it came to the exports of the commodities that were produced from cereal crops, wheat, maize, and rice contributed 45%, 32%, and 9% respectively. Simultaneously, soybean contributed 28% of oil production (FAO, 2022). These crops are of keen importance to address the food security issue. Hence, the global research developments can be examined to update and for self-evaluation in fulfilling the current food demands. By delving deeply into a particular study’s academic history and knowledge structure, it is possible to identify the research themes, knowledge base, research frontiers, and hotspots worldwide. The extensive background of scientific investigations can be categorized using a range of distinct specialties, including institutions, collaborating authors, countries, co-occurring keywords, publications, hot research topics, cited references, and knowledge clusters. Leveraging the co-occurring keywords in articles from databases to conduct a literature clustering helps to reveal the knowledge structure and domains (Qian et al., 2019; Chen and Liu, 2020). These maps can be represented as networks to create a structure of these analyses known as bibliometric (or scientormetric) analysis (Ahmed et al., 2021; Azam et al., 2021; Hussain et al., 2023). Consequently, this review systematically interprets the intellectual history of a research topic or scientific literature through this novel approach. Moreover, it provides insight into new breakthroughs relevant for researchers, business investors, engineers, and the author or institutional collaboration knowledge structures and emerging research topics. This review article focusses on the use of UAVs in yield prediction (YP), and particularly with applications to wheat, maize, rice and soybean (WMRS) cropping systems: given its importance for food security at global scale.

**Figure 1:**
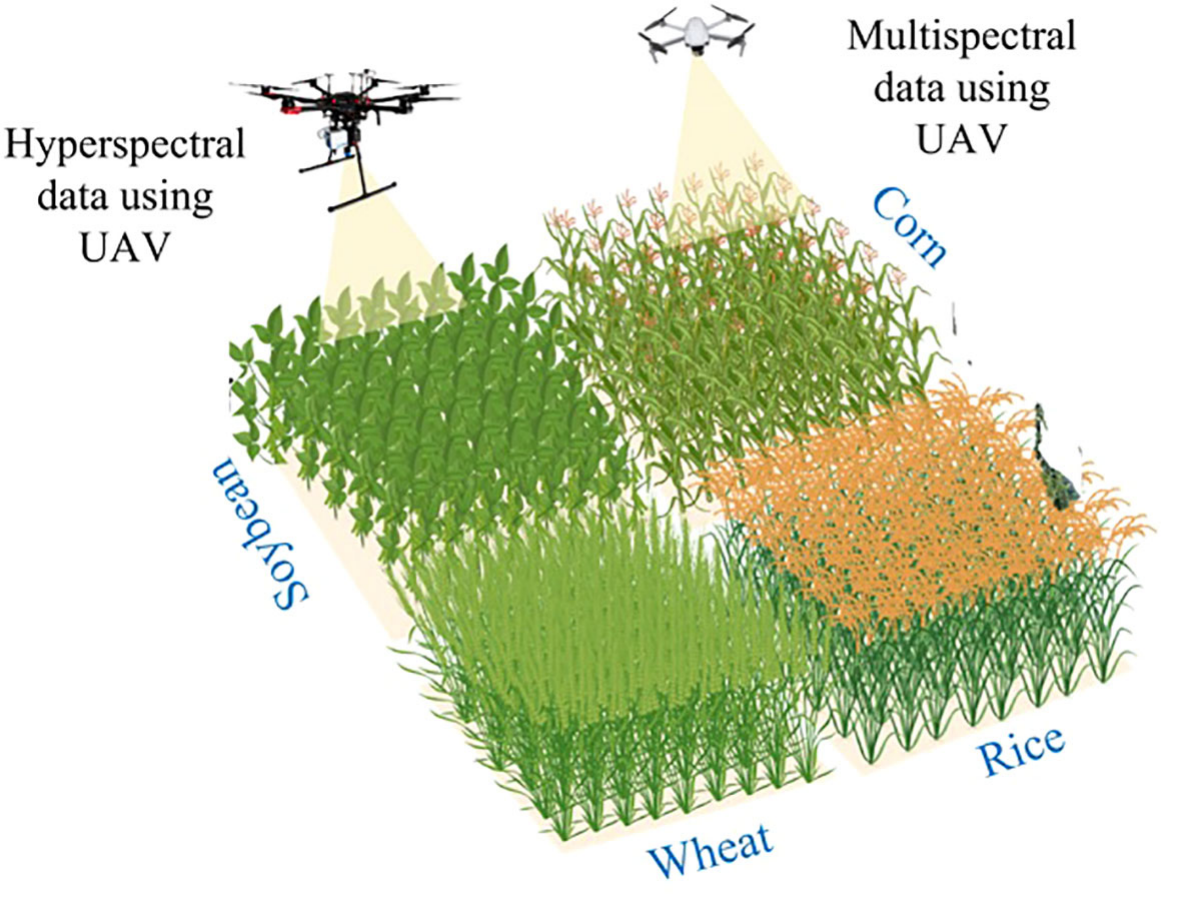
Pictorial view of the application of the UAV for crop yield prediction.

Numerous approaches are being made for WMRS’s sustainable production, protection, monitoring, and estimation, and one use is UAVs. YP analysis has been increasingly popular as big data technologies have advanced (Barnetson et al., 2020). Consulting firms and policymakers frequently employ such models when developing effective strategies (Astor et al., 2020). One of the most critical challenges in agriculture is grain yield regarding personal living standards and food security. The quantity of crop produced in a certain year is referred as the yield (Fischer et al., 2014). Several factors affect here, like genetic features, soil, weather, cultivation, and a wide range of varietals crop yields. Remote sensing (RS), a relatively new technique is expected to help calculate rice yield, especially on a regional scale. With the emergence of UAVs, a unique strategy for RS has been provided, and high spatiotemporal resolution imaging on a regional scale is now possible (Zheng et al., 2019). However, it wasn’t till 2000s that the UAV technology took off in the agricultural sector, thanks to ground-breaking innovations that made the technology more accessible, user-friendly, and affordable. Examples of agricultural-grade UAVs with sensors available in 2019 include the Matrice 100 (Dà-Jiang Innovations, Shenzhen, China) and MicaSense Red Edge MX (Seattle, Washington) as well as the Pix4D Fields (Lausanne, Switzerland) and hardware from Pix4D (Puget Systems, 2021) could be purchased for less than $30,000. In addition, using UAVs' sensors has facilitated the collection of visual data, which can now be analyzed through cloud-based computing services. This advancement has decreased the requirement for costly image processing software and hardware that formerly had to be present on-site. Notably, Pix4D Fields, a software developed by Pix4D in Lausanne, Switzerland, is an example of such cloud-based computing services. Typically, the acquisition of UAVs-based data involves several stages: the formulation of a mission plan, the collection of imagery during the operation, the subsequent stitching, processing, and extraction of the data, and ultimately, the uploading of the resulting data output to precision agriculture machines or for statistical analysis in the context of research. **Figure 1** demonstrates a conventional method for acquiring quantifiable and actionable data from multispectral and hyperspectral UAVs.

Additionally, satellites, manned airplanes, and handheld sensors can all be used for RS (Melesse et al., 2007). However, satellites’ drawbacks include lesser resolutions, cloud blockage, scheduling, and location issues. Manned aircraft can cover large areas, but the expense is overpriced. While handheld sensors are highly accurate, they have far smaller coverage areas than aerial RS. UAVs are appealing for agricultural applications due to their ability to efficiently cover large areas, unaffected by cloud interference, unrestricted by location or timing constraints, and reasonably cost-effective (Muchiri and Kimathi, 2022). However, some disadvantages of UAVs-based RS are data degradation due to lighting conditions, data collection’s tendency to occur close to solar noon, airspace restrictions, and poor weather grounding. Regulations regarding the usage of UAVs vary greatly throughout countries and often encompass limitations on velocity and elevation, nocturnal operations, visual range, proximity to airports, and densely populated regions. Additional issues to take into account during secure flight operations include the presence of other manned aircraft, prey birds, and disruptions in controller communication that could result in out of control.

Crop improvement through genetics and plant breeding and the use of precision technologies such as UAVs and remote sensors are potential solutions to meet this demand (Yang et al., 2017). Such technologies are vital for strategic management and can lead to specific breeding decisions and ensure the maximum agricultural outputs (Tilman et al., 2011). The technologies like UAVs in RS, enable rapid collection of phenotypic data with an efficient and non-destructive way for agronomists and plant breeders (Yang et al., 2017). Examples include estimating leaf color (Graham et al., 2009), lodging (Zhao et al., 2019), plant height (Han et al., 2018), stand count (Zhao et al., 2018), canopy cover (Lee and Lee, 2011), fruit count (Dorj et al., 2017) and flower count (Adamsen et al., 2000). The spectral sensors can be used to calculate yield (Shanahan et al., 2001), leaf area index (Boegh et al., 2013), leaf chlorophyll content, indirect leaf nitrogen content, and plant biomass. Finally, thermal sensors gather information to calculate canopy temperature, stomatal conductance, plant water potential and water use efficiency (Santesteban et al., 2017).

Phenotyping estimations rely significantly on the duration of sensing, and as crops reach maturity, phenotyping assessments improve in accuracy. In most cases, the difference between the terrain model and the surface model must be calculated to predict crop height; this yields the so-called digital crop model (Han et al., 2018). Low-resolution cameras (3 megapixels) cannot catch the fine details of complex crop surfaces, hence only cameras with superior resolution (>10 megapixels) should be utilized. Additionally, it has been demonstrated that the “Scale Constraint” function of some imaging processing software and ground control points improves crop height estimates. Refraining from conducting sensing activities on days with wind speeds over 1-10 kilometers per hour is advisable (Sziroczak et al., 2022). This precaution is recommended due to the propensity of wind to induce movement in plants and result in image blur, hence diminishing the precision of crop height estimation.

### 1.1 Background for yield assessment

Numerous parties rely on crop output and quality estimates, including consultants, producers, academics, insurance agents, commodities merchants, governments, and non-governmental organizations (Rembold et al., 2013). This data helps to determine how much crop insurance to purchase, how much to deliver, when to harvest to maximize quality, how much space is needed for storage, and how much money will be needed. Traditionally, managers have relied on historical yield data and seasonal variables to inform their yield and quality assessments for the remaining season (Raun et al., 2001). In contrast, the ultimate outcomes of crop production and the resulting quality are characterized by a lack of predictability and often subject to factors such as crop genetics, weather conditions, soil composition, proficiency, and choices made in crop management. The assessment of crop production and quality through the utilization of UAVs technologies commonly depends on data acquired from color and spectral sensors. The utilization of UAVs imagery, particularly in conjunction with machine learning (ML) techniques, currently holds the capacity to enhance assessment precision and potentially diminish or eradicate the need for terrestrial surveys. The accurate estimation of crop yield and quality is contingent upon the ability to effectively sense time, as the accuracy of estimation tends to improve as the crop progresses through its life cycle (Ballester et al., 2017; Zhou et al., 2017).

The green normalized difference vegetation index (GNDVI) yielded more precise biomass estimations at the stages of anthesis and full crop development than at earlier stages of development (Ostos-Garrido et al., 2019). According to other studies, it has been observed that the GNDVI yielded more accurate estimations of crop production at the early stage (5 weeks) of a crop’s growth cycle (Wahab et al., 2018). Consequently, great care must be taken in the selection of factors (color component and saturation indices), as this choice greatly influences the efficacy and precision of crop yield and quality estimates using spectral reflectance obtained at peak sites.

However, environmental factors and crop water stress are likely to hinder the performance reliability of spectral indices where NDVI (Normalized Difference Vegetation Index) and RENDVI (Red Edge Normalized Vegetation Difference Index), based on physiological behavior measured in lab conditions rather than actual meteorological variations under field environments for predicting wheat production accurately (Hassan et al., 2019). Consequently, forthcoming investigations will examine these elements within stress-controlled experiments to cultivate further understanding. Incorporating correction factors for quality and yield assessments may be seen as a potential approach to enhance the accuracy of spectrum consumption measurements in indices. The application of ML, namely artificial neural networks, to spectral bands has demonstrated potential (Sarkar et al., 2018). For instance, these protocols have successfully predicted the grain protein content of rice (*Oryza sativa*) and the total soluble solids content of grapes (*Vitis*). Relatively inexpensive color cameras have been utilized to capture color characteristics, proving to be a dependable method for generating accurate yield estimations (Kefauver et al., 2017). This approach offers a feasible alternative to utilizing more expensive sensors. However, previous investigations that have employed color cameras have yielded unsatisfactory outcomes (Bura et al., 2018). This could be attributed to the inadequate development of yield estimation protocols, or the studies conducted under unfavorable environmental conditions (Bura et al., 2018). In conclusion, numerous studies have been undertaken to estimate crop production (Zhou et al., 2017; Bura et al., 2018; Hassan et al., 2019; Zheng et al., 2019). However, numerous studies have shown the concerns regarding accuracy, reliability, and scalability for the analysis of the UAVs data for different crop phonemics based studies.

#### 1.1.1 Accuracy

##### 1.1.1.1 Sensor restrictions

The UAVs usually have thermal, multispectral, or hyperspectral sensors (Fei et al., 2023). Even though these sensors can record much information about crops, they might not be precise enough for analyzing individual cereal crops, especially in environments with varying lighting and weather conditions (Lee et al., 2010).

##### 1.1.1.2 Weather and flight stability

Cloud cover, wind, and fluctuating sunlight are external elements that might add inaccuracies into sensor data (Aasen et al., 2018), impacting YP’s accuracy.

##### 1.1.1.3 Data quality

UAVs gather information at different speeds and altitudes, which could cause inconsistencies in the resolution and quality of the data collected from the sensors (Pádua et al., 2017). YP and analyses that rely on this ambiguity may be imperfect.

#### 1.1.2 Reliability

UAVs express difficulty flying in unstable air and are vulnerable to bad weather like wind, rain, and fog, which can delay data collection and even equipment damage (Ubina and Cheng, 2022). This unpredictability may make YP models less reliable (Müller et al., 2016).

##### 1.1.2.1 Data processing and integration

UAVs produce massive amounts of data that must be processed correctly to conduct real-time analyses (Guimarães et al., 2020). Because of inherent variances in data structure and format, integrating UAV data with other data sources, including weather, soil, and ground truth measures is not easy (Samaras et al., 2019).

#### 1.1.3 Scalability

##### 1.1.3.1 Field coverage

UAVs have trouble collecting data at scale due to their short flight times and short battery lives, which limits their capacity to survey vast cereal crop fields in a single flight (Mohsan et al., 2023).

##### 1.1.3.2 Data storage, integration and management

For analyses that span several growing seasons or include long-term monitoring, it is necessary to have a robust infrastructure to store and manage massive datasets acquired by UAVs (Nabwire et al., 2021). When applied to new locations with varied soil types, temperatures, and crop types, ML models built on data from one field or area might not perform as well as when trained on data from another (Benos et al., 2021). Hence, it limits the models’ ability to be applied at various agricultural scales.

#### 1.1.4 Addressing challenges

Diverse methods can be considered to overcome these previously mentioned difficulties.

##### 1.1.4.1 Sensor calibration and data fusion

The application of advanced filtering techniques and regularly calibrating sensors can help to increase the quality of data (Concas et al., 2021). A more complete picture of crop conditions and improved yield projections can be achieved through data fusion and integration (Ahmad et al., 2022), which involves combining data from UAV with data from other sources (such as satellite imaging and ground-based sensors).

##### 1.1.4.2 Hybrid machine learning models

By integrating ML with more conventional statistical approaches, scientists can create more robust models and generalize to new datasets (Elavarasan et al., 2018).

##### 1.1.4.3 Improved technology for UAV

Longer flight times and more automated flight controls are just two examples of how UAV technology is constantly evolving to better cover more ground and collect more data (Chaurasia and Mohindru, 2021).

##### 1.1.4.4 Data management solutions with cloud computing

Using the cloud to store and analyses data can solve scalability problems and make data analysis more effective (Delgado et al., 2019). The background reveals proof of adaptation of the multiple approaches for YP in different cereal crops.

This study employs UAVs for YP and provides a bibliometric analysis of existing research conducted at WMRS crops. The WOSCC extracted pertinent scientific information published between 2005 and 2023 in high caliber publications. VOSviewer was used for co-occurrence, co-citation, and co-authorship analyses. The following are the goals of the current study: (a) To establish a knowledge structure for WMRS crops using UAVs for the YP (b) To evaluate the use of ML, different sensors and limitations for WMRS crops. (c) To identify the knowledge gaps and research frontiers for YP in WMRS crops employing UAVs. The article’s structure includes an introduction to WMRS crops and their importance, publication collection, a description of the results of the bibliometric analysis, review on WMRS crops, constraints and prospects for future study, and contribution of the current study for the scientific community.

## 2 Retrieval of publications’ information and outline for bibliometric methods

### 2.1 Publications’ information retrieval from web of science

The most reliable literature-indexing site is the Web of Science (WOS), which includes scientific, social, health, and economic information. Therefore, it is acknowledged that the global WOS is the ideal source for gathering data for bibliometric analysis (Chen and Liu, 2020). The pertinent information was gathered from the WOS core collection (WOSCC) databases. Many iterations were employed to find the best keyword search code to download the most pertinent RS-related publications for YP in WMRS crops utilizing UAVs. **Table 1A** shows a list of more specific keyword codes that can be used to probe the WOS database systematically. The most useful search keywords were as follows: (“UAV” OR “multispectral” OR “hyperspectral” OR “RGB”) (Topic) and (“yield prediction” OR “yield estimation”) (Topic) and (“maize” OR “rice” OR “wheat” OR “soybean”). Be noted that the published articles were examined for the presence of words within their titles, abstracts, or keywords. All searched documents were validated for relevant material, and manually irrelevant articles were removed. The extraction process involved selecting solely peer-reviewed, original research publications that were published in the English language. The data acquiring period spanned from January 1, 2000, to August 3, 2023 (**Table 1B**), and the relevant scholarly material and the scope of inquiry were restricted to science and technology.

**Table 1:** Selection of optimized keywords for WOS publications’ information, and VOSviewer values and parameters for bibliometric examination for yield prediction in WMRS crops utilizing UAVs.

(A) Selection of optimized keywords for WOS publications’ information			
No.	Searching code	Results	Quality
1	(“hyperspectral” OR “reflectance” AND “wheat” AND “yield prediction”)	50,159	very generic, very rough, highly irrelevant
2	(“UAV” AND “wheat” AND “yield prediction” OR “yield estimation”)	3,478	Very rough, highly irrelevant
3	(“UAV” AND “wheat” AND “yield prediction”)	75	yet irrelevant, Improved,
4	(“UAV” AND “wheat” AND “yield prediction” OR “yield estimation”) (Topic) and (“UAV” AND “wheat” AND “yield prediction” OR “yield estimation”)	448	Very generic and moderately irrelevant
5	(“UAV” AND “wheat” AND “yield prediction” OR “yield estimation”) (Topic)	1907	Improved, yet irrelevant
6	(“UAV” OR “multispectral” OR “hyperspectral” OR “RGB”) (Topic) and (“yield prediction” OR “yield estimation”) (Topic) and (“maize” OR “rice” OR “wheat” OR “soybean”)	226	Highly improved, fully relevant
(B) VOSviewer values and parameters for bibliometric examination of advanced research			
No.	Parameters	Definition	
1	Time slicing	2001-01-01 to 2023-08-03	
2	Term source	Abstract, keywords, title, author, and keywords plus	
3	Node type	Country, institution, cited author, Author, cited journal & keywords cited reference.	
4	Selection criteria	Top 15%	
5	Links	Default	
6	Visualization	Cluster static and combined network views are displayed	

### 2.2 Work flow for the study

**Figure 2:**
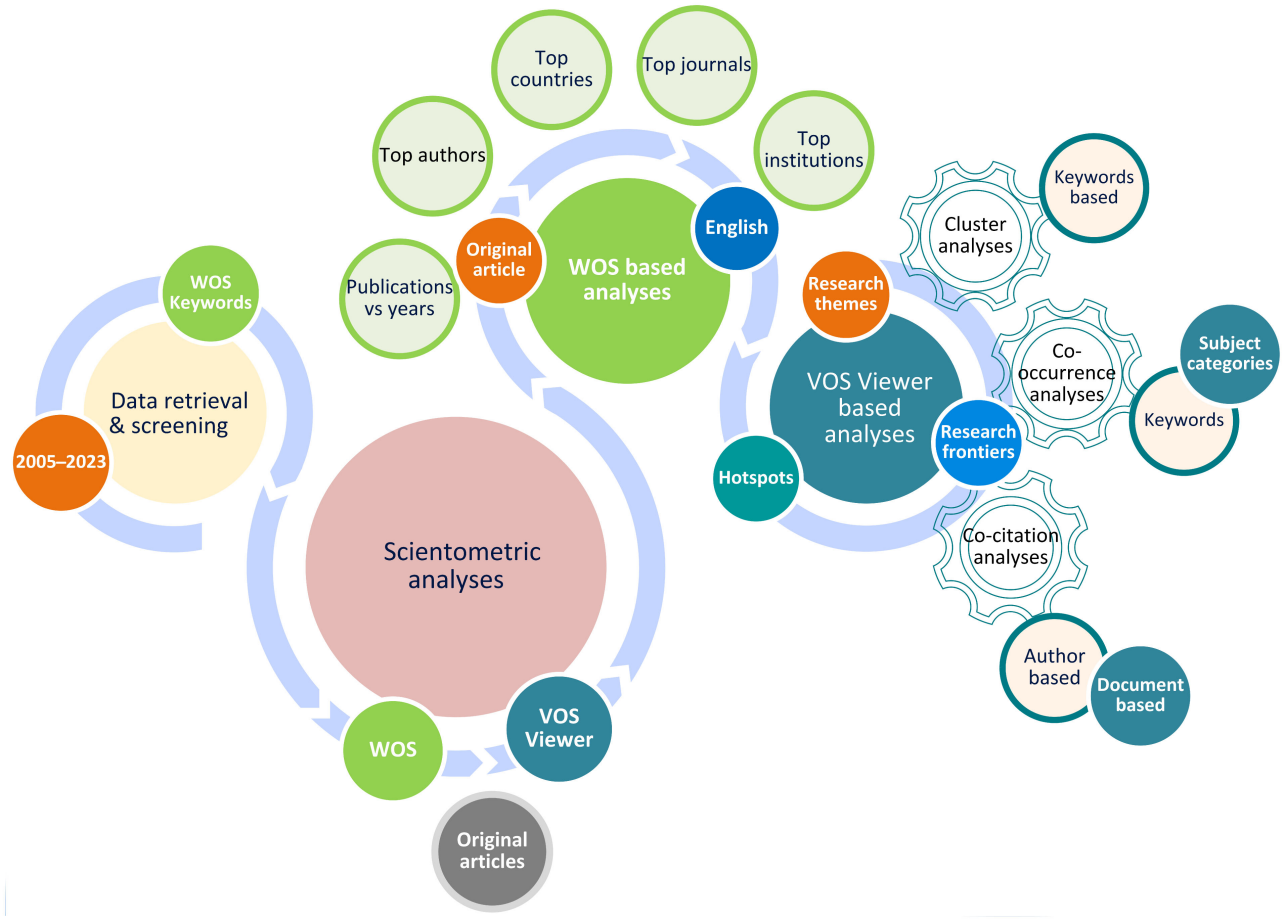
Outline for the data retrieval and VOS-viewer based bibliometric analysis.

An overall number of 226 original research publications were retrieved using the methods described above. The entire document and all referenced materials were saved as “Tab-delimited” as the preferred file type. The schematic shows the actions performed to carry out the investigation in **Figure 2**.

### 2.3 VOSviewer based bibliometric analysis

A map or network analysis and the visualization of scientific literature are examples of more advanced bibliometric analysis skills. Nees Jan van Eck and Ludo Waltman developed a robust program called VOSviewer (Version 1.6.18) for investigating and visualizing bibliometric networks (Van Eck and Waltman, 2010). It makes it possible to do analyses such as co-citation analysis, cluster analysis, and bibliometric mapping, all of which highlight the current research collaborations and patterns. The scientific community relies heavily on VOSviewer to visualize and project their data. The scientific community can learn more and get the program at http://www.vosviewer.com.

As per the specified parameters, utilizing VOSviewer facilitated the execution of co-citation analysis and keywords co-occurrence analysis. These analyses generated networks visually representing the co-citations seen among authors, documents, journals, and keywords. Furthermore, these networks provided insights into prominent study themes and emerging areas of investigation. In conclusion, the relevant data and mapping networks were thoroughly analyzed, and the results of the visualization inquiry in the present research study were presented and discussed.

## 3 Interpretation and discussion of the bibliometric results

### 3.1 Publications’ information analyses retrieved from web of science

**Figure 3:**
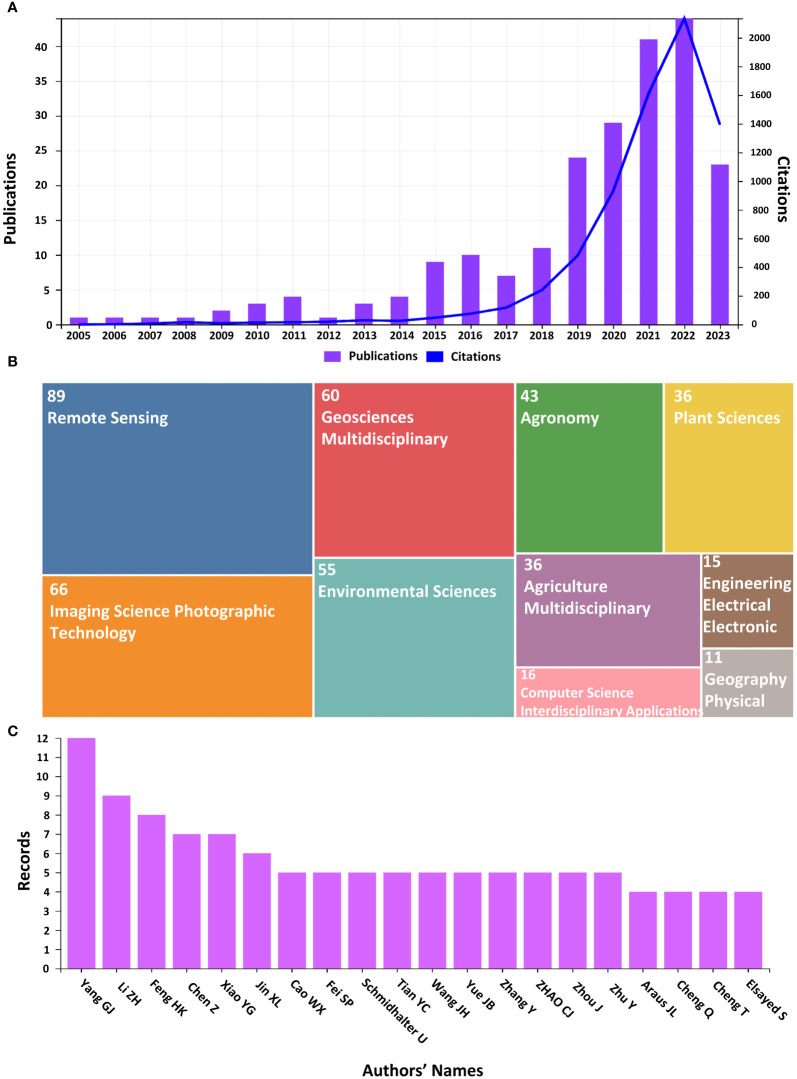
**(A)** Illustrates the number of publications and citations, **(B)** Number of publications according to subject categories, **(C)** Number of records for different authors.

The distribution of citations and matching publications in the domain of YP using RS in WMRS crops utilizing UAVs throughout the years 2005-01-01 to 2023-08-03 is shown in **Figure 3A**. It can be seen that over the first ten years (from 2005 to 2011), there was a prominently gradual increase in the number of notable publications. In 2012-2014 there was a decrease in publications. However, from 2014 to onward, there is a continuous and gradual increase in publications. In total, 44 research were published in 2022, which is the highest. Of the whole, 60.25% of the total publications are published from 2019-2022, 19.89% only in 2022, while 2023 has contributed 10.52% so far. The concerns about the research dramatically increased starting from 2019 to onward. This can be related to shifting funding preferences or research priorities during that time. The number of citations for publications remains gradually growing, while it has shown a fall in 2023, which might be due to the incompletion of this year. The highest citations (2291) have been reported in 2022, reflecting the impact of the research in this field. This upsurge could be attributed to developments in ML and UAV technology, which facilitate the conduct of creative and significant research.

The WOS based subject category area (**Figure 3B**) shows the publication distribution in the top 10 scientific study areas. Where “Remote Sensing” is leading than all others, having 39.04% (89 Articles) of total articles. While Agriculture, Imaging Science, Photographic Technology, Geosciences Multidisciplinary, Environmental Sciences, Agronomy and Plant Sciences share 89, 66, 60, 55, 43 and 38 publications, respectively. This diversity draws attention to the research’s interdisciplinary nature. The more influenced 15 authors for the YP in WMRS crops utilizing UAVs are ranked in **Figure 3C** where Yang GJ ranked at top with the maximum number of records in the acquired publications’ dataset. Given their prominence, it appears that they have given this field of study a lot of attention in their work.

**Table 2A** lists the top 15 journals in which the most articles relevant to the research area of YP in WMRS crops utilizing UAVs are published, along with the number of publications and their percentage contribution. **Table 2B** lists the top 15 research institutions for YP in WMRS crops utilizing UAVs. **Supplementary Tables S1, S2** indicate the 15 most important countries and prolific writers with the most studies in the specified research subject, respectively. **Supplementary Table S3** shows the distribution of the top fifteen funding agencies involved in relevant publications collected from the WOS in YP research utilizing UAVs. This systematic methodology provides insightful information and enables researchers to narrow down areas of potential studies for future.

**Table 2A:** The 15 best journals for publishing research on yield prediction in WMRS crops utilizing UAVs.

No.	Journals	Records	% of total
1	REMOTE SENSING	46	20.354
2	FRONTIERS IN PLANT SCIENCE	15	6.637
3	COMPUTERS AND ELECTRONICS IN AGRICULTURE	13	5.752
4	PRECISION AGRICULTURE	11	4.867
5	IEEE INTERNATIONAL SYMPOSIUM ON GEOSCIENCE AND REMOTE SENSING IGRASS	8	3.540
6	AGRICULTURE BASEL	7	3.097
7	AGRONOMY BASEL	7	3.097
8	PLANT METHODS	7	3.097
9	SENSORS	6	2.655
10	AGRICULTURAL AND FOREST METEOROLOGY	5	2.212
11	BIOSYSTEMS ENGINEERING	5	2.212
12	DRONES	5	2.212
13	PROCEEDINGS OF SPIE	5	2.212
14	FIELD CROPS RESEARCH	4	1.770
15	ISPRS JOURNAL OF PHOTOGRAMMETRY AND REMOTE SENSING	4	1.770

**Table 2B:** The 15 best institutions for publishing research on yield prediction in WMRS crops utilizing UAVs.

No.	Affiliations	Records	% of total
1	BEIJING ACADEMY OF AGRICULTURE FORESTRY SCIENCES BAAFS	19	8.407
2	MINISTRY OF AGRICULTURE RURAL AFFAIRS	19	8.407
3	CHINESE ACADEMY OF AGRICULTURAL SCIENCES	17	7.522
4	CHINA AGRICULTURE UNIVERSITY	12	5.310
5	CHINESE ACADEMY OF SCIENCES	12	5.310
6	INSTITUTE OF CROP SCIENCES CAAS	10	4.425
7	NANJING AGRICULTURAL UNIVERSITY	10	4.425
8	ZHEJIANG UNIVERSITY	9	3.982
9	UNITED STATES DEPARTMENT OF AGRICULTURE USDA	8	3.540
10	WUHAN UNIVERSITY	8	3.540
11	UNIVERSITY OF MISSOURI COLUMBIA	7	3.097
12	UNIVERSITY OF MISSOURI SYSTEM	7	2.097
13	UNIVERSITY OF NEBRASKA LINCOLN	6	2.655
14	UNIVERSITY OF NEBRASKA SYSTEM	6	2.655
15	EGYPTIAN KNOWLEDGE BANK EKB	5	2.212

### 3.2 Citations’ analyses

When a third author or document cites two or more authors or documents simultaneously, this is called a co-citation link (Chang et al., 2015). VOSviewer uses three main types of co-citation analyses to show how documents, writers who cite each other, and journals are connected and how they map to each other. Co-citation analysis is a good way to determine how many connections between journals, authors, and papers. It builds a mapping framework and tracks how scientific research areas change over time (Behrend and Eulerich, 2019).

#### 3.2.1 Co-citation analysis for documents

**Figure 4:**
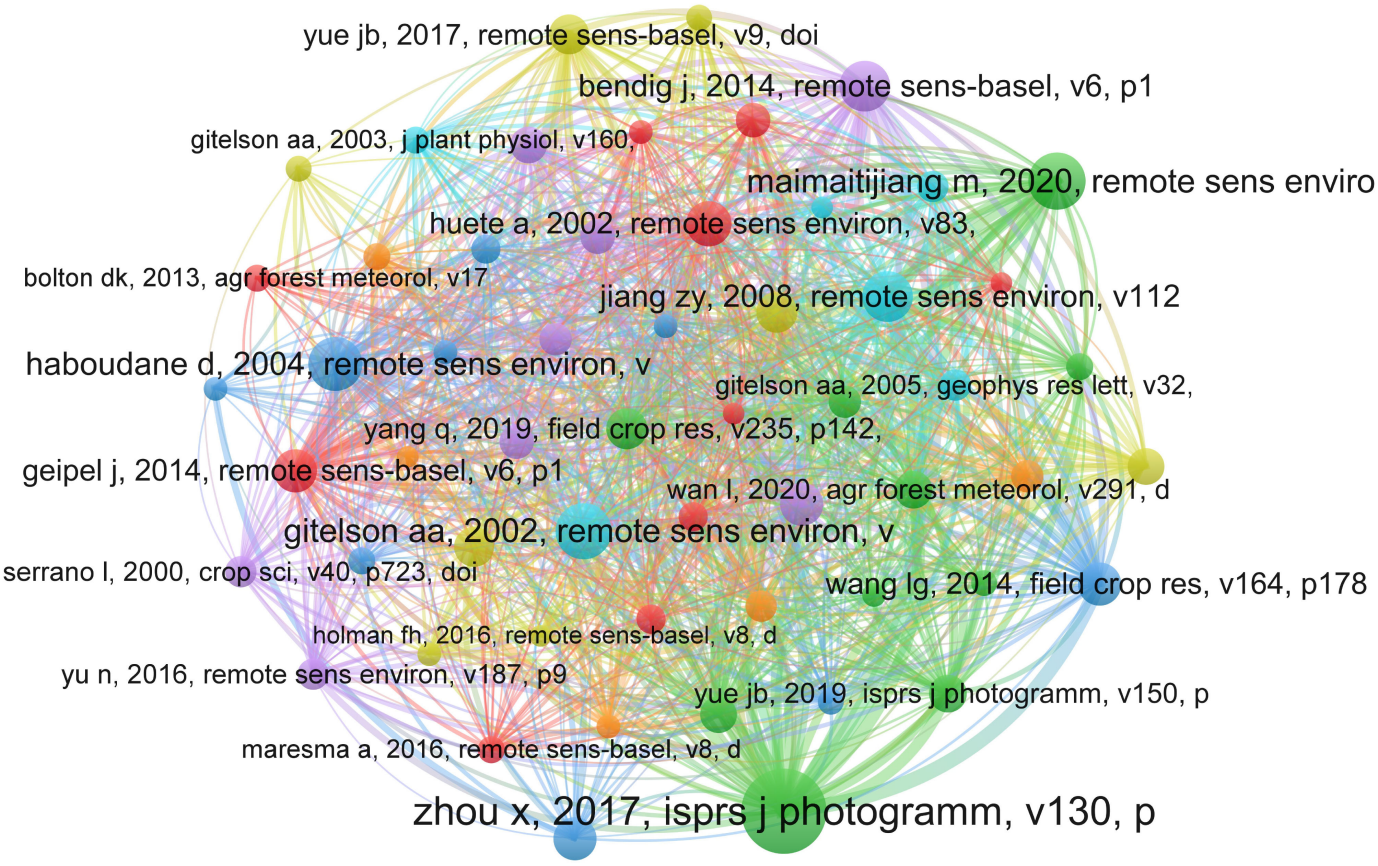
The document co-citation network visualization map for research on yield prediction in WMRS crops utilizing UAVs.

The documents or articles are the most important parts of the knowledge repository or database in the area of predicting crop yields using UAVs for WMRS crops. Reference co-citation analysis, also called document co-citation analysis, is a good way to look at how a study area has grown and changed over time (Liao et al., 2018). After the scientometric study in VOS viewer was done, a network for displaying cited documents was made (**Figure 4**). Every node represents a reference or article cited, and the links between the nodes demonstrate how the mentioned references and articles relate to one another. Larger nodes represent more essential documents, and documents often referenced by other documents are close to each other. **Figure 4** shows the most important works completed in this field are (Zhou et al., 2017) (Haboudane et al., 2004), and (Maimaitijiang et al., 2020).

#### 3.2.2 Co-citation analysis for authors

**Figure 5:**
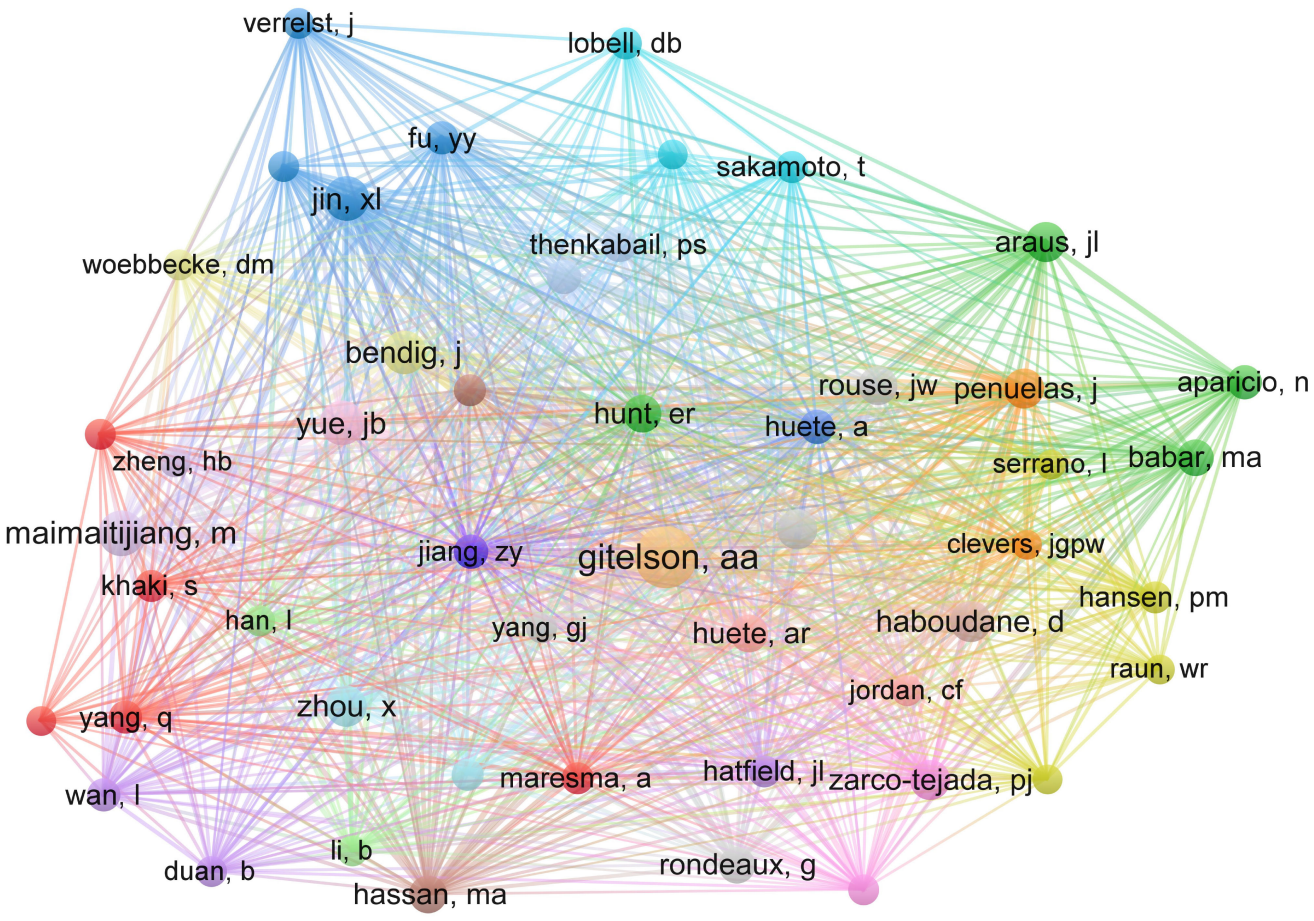
The authors’ co-citation network visualization map for research on yield prediction in WMRS crops utilizing UAVs.

The distribution of authors with more citations in a particular field of study is also examined by the co-citation analysis for the author, which is used to identify the most productive writers in that subject. The co-citation analysis also makes the visualization of associated writers’ subject areas and research interests feasible. The visualization network that was produced after the author’s co-citation analysis of the study into YP in WMRS crops using UAVs is shown in **Figure 5**. The nodes represent authors, the connecting lines between two nodes indicate their relationship regarding co-citations. An author’s importance is increased when a node gets larger because that particular author makes more citations.

Similarly, the distance between two successive nodes or writers is inversely associated with the amount that one author is cited by the other. The authors’ areas of interest will be more strongly correlated with the size of the distance between the nodes. Author co-authorship analysis confirms the extensive analysis of the visualization network, which shows that most authors collaborate to a very high degree. It offers vital insights into the organization and movement of a research field, aiding researchers in comprehending significant contributors, patterns, and possibilities for collaboration.

The 15 best most co-cited authors are listed in **Table 3A** along with their respective authors, counts of citations, years of citation counts, and rankings based on the times their scientific literature have been cited. According to the data, the aforementioned authors’ work made a significant contribution to the field of YP in WMRS crops utilizing UAVs, making them significant participators to the future growth of YP research. According to the findings, the authors (Zhou et al., 2017), (Maimaitijiang et al., 2020), and (Yue et al., 2019) were the most prolific in the research field,

**Table 3:** The 15 best co-cited authors and top fifteen keywords for research on yield prediction in WMRS crops utilizing UAVs.

Table 3A The 15 best co-cited authors for publishing research			Table 3B Top fifteen keywords in the domain of yield prediction		
Sr. No.	Count	Cited Authors	Ranking	Counts	Keywords
1	68	ZHOU, X	1	110	Vegetation indices
2	63	MAIMAITIJIANG, M	2	84	UAV
3	63	YUE, JB	3	60	Wheat yield
4	59	JIN, XL	4	59	Yield estimation
5	50	BENDIG, J	5	46	Biomass estimation
6	49	HABOUDANE, D	6	44	Yield prediction
7	47	ARAUS, JL	7	43	Winter-wheat
8	46	TUCKER, CJ	8	43	Grain-yield
9	45	ZARCO-TEJADA, PJ	9	40	Remote sensing
10	45	PENUELAS, J	10	38	Leaf-area index
11	43	ROUSE, JW	11	27	Corn yield
12	41	HASSAN, MA	12	26	Machine learning
13	40	HUETE, AR	13	26	Chlorophyll content
14	39	RONDEAUX, G	14	25	Precision agriculture
15	39	BABAR, MA	15	25	Maize yield

#### 3.2.3 Citations analysis for documents

**Figure 6:**
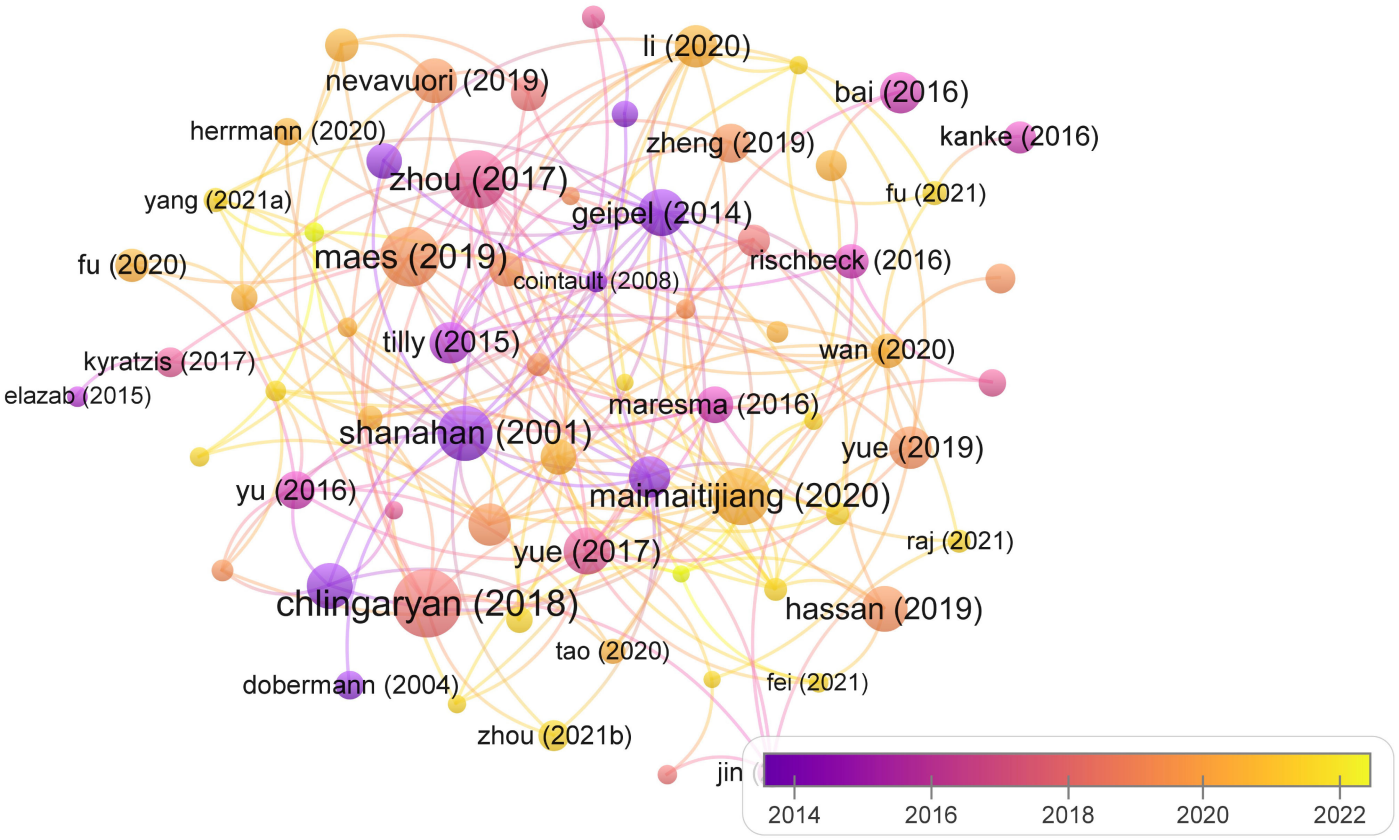
Citations’ analysis of documents for yield prediction in WMRS crops utilizing UAVs.

Citation analysis for authors represents a bibliometric approach employed to assess the impact and influence of an individual author’s scholarly output within the academic and relevant research field. This method entails comprehensive scrutiny of the frequency with which an author’s publications have garnered citations in the works of other researchers. Through the examination of citation patterns, this analytical method provides valuable insights into an author’s contributions to their field of study, the level of recognition their research receives, and the extent of their influence within the scholarly and academic community. **Figure 6** visualizes the articles with more than 20 citations from different years. The size of nodes represents the highly cited documents, like (Chlingaryan et al., 2018), (Zhou et al., 2017), (Maes and Steppe, 2019), and (Shanahan et al., 2001) are most prominent with superior size of nodes.

### 3.3 Keywords’ co-occurrence analysis

**Figure 7:**
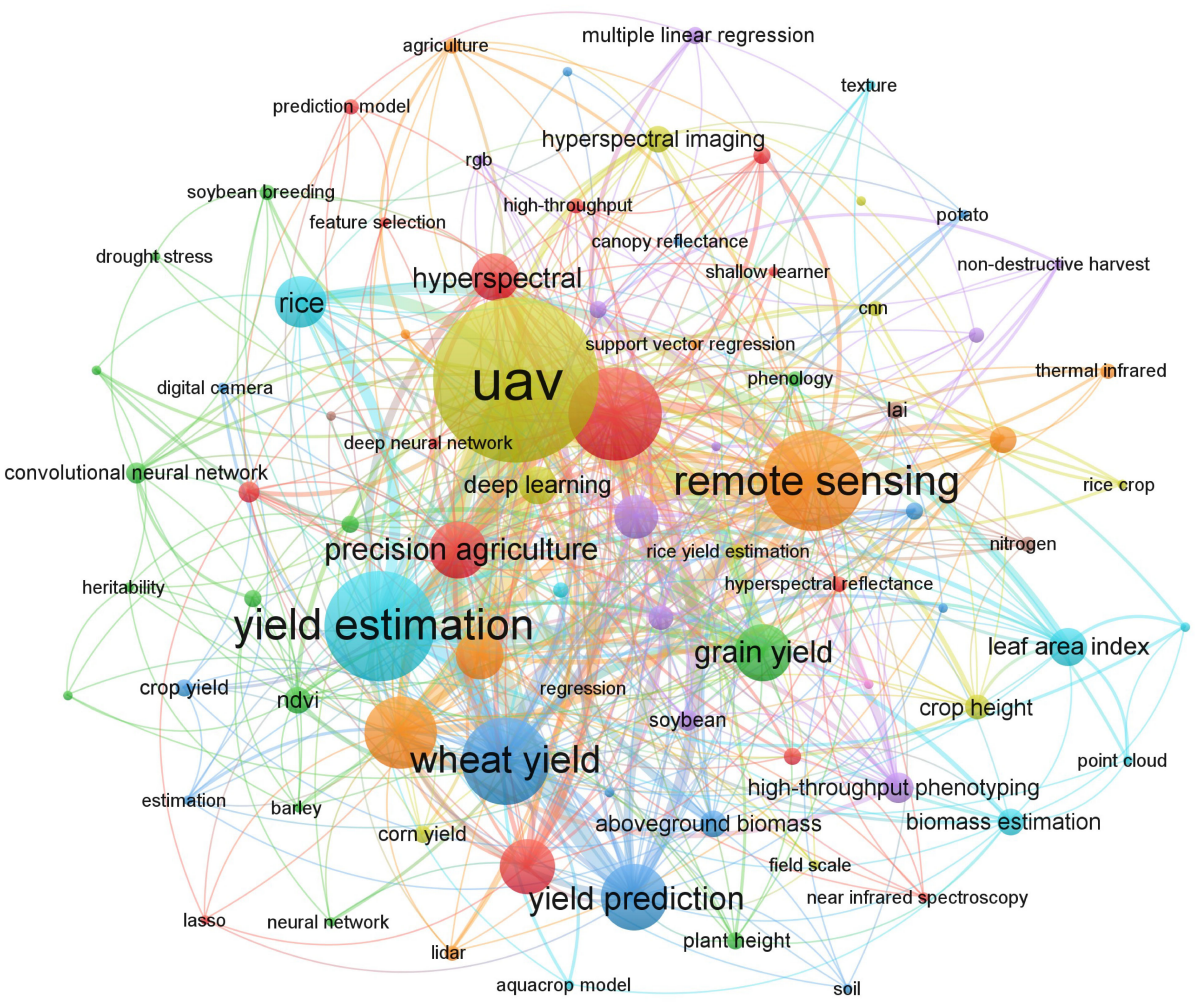
Keywords’ co-occurrence analysis for yield prediction in WMRS crops utilizing UAVs.

In a scholarly context, keywords serve as descriptors that elucidate the precise subject matter or overarching category to which an article pertains. Additionally, they encapsulate the primary information summarized within research papers. In essence, keyword co-occurrence analysis can be employed to discern prevailing focal points and emerging research frontiers. The most rapidly increasing citation rates for individual keywords indicate perennially popular issues or promising avenues for future study. **Figure 7** is a visual representation of the VOSviewer keyword co-occurrence analysis results. Nodes represent the keywords, and the size of the nodes represents the frequency with which they occur together.

**Table 3B** presents a ranking of the top fifteen keywords in the domain of YP in WMRS crops utilizing UAVs. These keywords have been ordered based on their occurrence frequency. The keywords demonstrating the most pronounced co-occurrence frequencies, along with their respective counts, include Vegetation indices (110 occurrences), UAV (84 occurrences), Wheat yield (60 occurrences), and Yield estimation (59 occurrences). Citation frequency analysis offers a concise insight into the prevalence of frequently employed keywords within a specified timeframe. This analysis allows for the temporal depiction of these terms, drawing attention to the time frame in which they were employed and referenced most frequently.

### 3.4 Co-authorship analysis

Cooperative analysis represents a multifaceted approach that delves into various tiers of investigation, spanning from the broad macro scale to the intricate micro level. Its objective is to unveil the intricate distribution patterns characterizing the collaborative dynamics within scientific research. In this specific context, the formidable tool of choice is VOSviewer, a sophisticated visualization software adept at illuminating the intricate web of collaborative relationships among institutions, countries/regions, and authors operating in the specialized domain of YP in WMRS crops utilizing UAVs. The calculation of the depth of collaborative involvement utilizes both fractional and full counting approaches, where full counting assigns equal weight to each participant.

Within the analytical framework of literature studies, two distinctive counting methodologies come into play: fractional and full counting. In the full counting mode, each contributor is endowed with an equal weight of 1, which is symmetrically distributed in quantifying the depth of their collaborative involvement. VOSviewer, as the computational engine, undertakes the intricate task of computing scores that gauge the interrelationships among knowledge units. Employing the precision of the association strength algorithm, it harmonizes and standardizes the raw data. The result concludes in creating an enlightening visualization map of the literature, encompassing distance-based and graph-based representations, thereby offering a comprehensive insight into the complex web of scholarly interactions.

#### 3.4.1 Co-authorship analysis for authors

**Figure 8:**
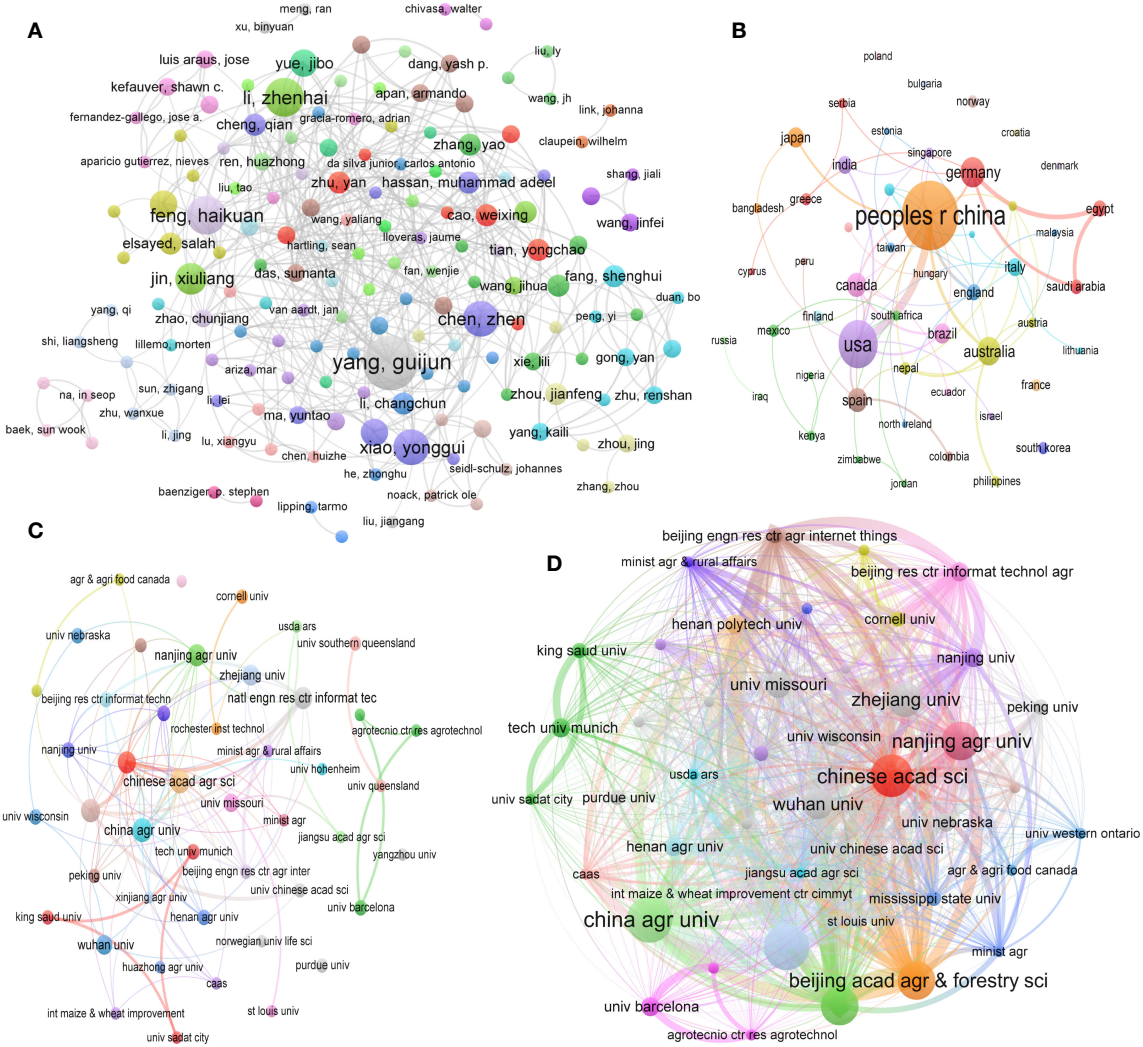
**(A)** Co-authorship analysis of authors, **(B)** Co-authorship analysis of regions/countries, **(C)** Co-authorship analysis of institutions, and **(D)** Coupling analysis of institutions for yield prediction in WMRS crops utilizing UAVs.

The quantification of an author’s productivity within a specific domain is a pivotal metric in assessing their impact within that particular field. The analysis of author collaboration serves as a valuable tool for investigating YP in WMRS crops utilizing UAVs and the associated social networks of cooperation. In **Figure 8A**, the study presents the author collaboration network within the domain of yield predictionYP in WMRS crops utilizing UAVs, focusing on authors who have contributed for at least two articles.

As depicted in **Figure 8A**, the collaboration landscape in this field exhibits a persistent spirit of cooperation and manifests a small-world effect. It is evident that each academic group maintains the capacity to engage in direct or indirect collaboration with other scientific research teams, illustrating the continuous transmission of information within the network. Notably, within specific clusters, the trajectory of YP in WMRS crops utilizing UAVs is influenced by high-impact authors, such as Yang Guijun, Li Zhenhai, Feng Haikuan, Chen Zhen, Xiao Yonggui and several others (**Figure 8A**).

#### 3.4.2 Co- authorship analysis for regions/countries

**Figure 8B** visually represents international cooperation among countries and regions over time, incorporating dynamic elements. In this visualization, the size and color of nodes correspond to the volume of research documents produced and the average publication year within each respective country or region. Furthermore, the thickness of the connecting lines between nodes indicates the level of collaboration between these entities, with thicker links representing more significant cooperation. By analyzing **Figure 8B**, it is evident that China and the United States are the leading countries in deploying UAVs for YP in WMRS crops. Countries such as Germany, Australia, Spain, Japan, Saudi Arabia and Canada have also actively engaged in this field of research.

It is worth highlighting that China, owing to its remarkable research productivity, has forged robust collaborative ties with Unites States for YP in WMRS crops utilizing UAVs. Looking ahead, both the China and United States are poised to emerge as principal players in the field of RS. Progress in advancing RS for YP in these two nations is of global significance, given the far-reaching benefits it brings. The advancements and collaboration between China and the United States, the two greatest developed and emerging nations in the world, significantly impact the Asia-Pacific region and the global scene. Motivated by same goals including guaranteeing food security and reducing the impact of sustainable crop production, both countries have agreed to make use of crop production innovations.

#### 3.4.3 Co-authorship analysis for institutions

The examination of institutional cooperation yields valuable insights into organizations and groups that make significant contributions within a particular field. This analysis serves as a foundational step towards fostering enhanced future collaboration for organizations with 3 documents as co-authorship. To effectively depict the distribution of organizations and their collaborative relationships, this study employs VOSviewer, a visualization tool, to represent the network of institutional collaboration within the domain of RS for YP in WMRS crops utilizing UAVs, as presented in **Figure 8C**.

Each node’s size in the below illustration represents the total number of scholarly articles published by that institution. The thickness of the connecting lines represents the degree of collaboration between universities. Nodes sharing the same color signify a higher level of cooperation than nodes with distinct colors. This analysis reveals notable collaborative connections among universities and colleges. For instance, the Chinese Academy of Sciences with China Agriculture University, and Nanjing Agricultural University with Zhejiang University exhibit a pronounced cooperative association, as evidenced by their placement within the red and green clusters. Additionally, in this study, we observe a strong collaborative relationship between King Saud University and the University of Sadat within the red cluster. Several other clusters have also merged around productive institutions, thereby contributing to forming a diversified and expansive cooperation network within the landscape of RS for YP in WMRS crops utilizing UAVs.

#### 3.4.4 Coupling analysis for organizations

Organizations’ coupling analysis is a sophisticated bibliometric method used to examine the interconnections and relationships between different organizations or institutions within the context of scientific research and publication activities. It primarily focuses on quantifying and understanding the collaborative patterns and knowledge exchange dynamics among these entities, often in the context of specific research fields or disciplines. The insights gained from organizational coupling analysis can inform policymakers, funding agencies, and researchers about the structure and dynamics of collaborative networks in specific research domains. This information is valuable for fostering interdisciplinary collaboration, optimizing research investments, and advancing scientific progress by promoting effective knowledge exchange among organizations. The organizations with a minimum 3 documents together are illustrated in **Figure 8D**, Chinese Academy, Nanjing Agricultural University, China Agricultural University, and Beijing Academy of Agriculture and Forestry Sciences are more prominent in respective clusters and have shown a prominent connection in connecting lines and node size. By highlighting collaborative networks and influencing choices on research investments and interdisciplinary collaboration, the results of this analysis can direct researchers, politicians, and funding agencies.

## 4 Recent studies on WMRS crops for UAV-based yield prediction

In agricultural research, precise early YP is paramount for individual farmers and the broader agricultural sector. UAVs have demonstrated commendable efficacy in enhancing YP accuracy through the utilization of various data sources (Hassan et al., 2019; Zheng et al., 2019; Kumar et al., 2023). For instance, such accuracy has been achieved by harnessing metrics such as RGB-derived plant height and canopy cover (Chu et al., 2016), VIs (Gracia-Romero et al., 2017), and multispectral imagery (Kyratzis et al., 2017; Zheng et al., 2019; Su et al., 2023). It is worth noting that the temporal dimension plays a pivotal role in optimizing YPs, with multitemporal VIs, including those accumulated throughout the crop’s growing season, exhibiting superior performance compared to single measurements (Zhou et al., 2017).The investigations conducted using UAVs for YP have predominantly adjusted on experimental fields characterized by substantial variations in final yield due to factors such as nitrogen levels (Zhou et al., 2017), phosphorous concentrations (Gracia-Romero et al., 2017), or irrigation practices (Vega et al., 2015). However, it remains imperative to scrutinize the efficacy of these methods within the context of precision agriculture conditions, where variations are predominantly driven by edaphic and microclimatic factors and are comparatively less extreme. Furthermore, it is crucial to acknowledge that prevailing UAV-based YP studies have primarily revolved around developing empirical regression models. While these models serve the purpose of extrapolating yield estimates to encompass entire fields, it is imperative to recognize their inherent limitation – regression coefficients derived from one year’s data may not be transferrable to subsequent years at the same location, nor to different locations within the same year (Rembold et al., 2013).An alternative approach in this domain involves the estimation of yield based on crop growth models. For instance, the GRAMI growth model was successfully applied to rice YP using UAVs GRAMI growth model utilizing UAV-derived data (Kim et al., 2017). Nevertheless, it remains evident that substantial research endeavors are indispensable to delineate the optimal sensor configurations, flight timing, and refinement of crop growth models to harness UAVs information most effectively and reliably for YP purposes. Numerous studies conducted since 2020 are shown in **Table 4** as a summary for YP in WMRS crops utilizing UAVs.

**Table 4:** Brief summary of the studies conducted for yield prediction in WMRS crops utilizing UAVs.

Crop	Data type	Algorithmic/Mathematical expressions	Methodology	Accuracy	Country	References
Rice	MS	VIs, A	MSMA	R^2^ = 0.75, RRMSE= 0.15	China	(Su et al., 2023)
Maize	MS, R	VIs	OM, DSF	95.75%	Nepal	(Sapkota and Paudyal, 2023)
Rice	MS, WD	LD	MMDL	RMSE= 0.86, RMSPE=14, R^2^ = 0.65	Japan	(Mia et al., 2023)
Wheat	MS	VIs, RE, SA	LASSO-R	R^2^ = 0.73	Norway	(Shafiee et al., 2023)
Wheat	3-D PC	LAI	GF	R^2^ = 0.63	China	(Yang et al., 2023)
Maize	MS	VIs	GL, SVM, RF	88-89%	Lithuania	(Kavaliauskas et al., 2023)
Maize	MS	VIs	LR, KNN, RF, SVR, DNN	R^2^ = 0.71, RMSE = 1.08 Mg/ha	USA	(Kumar et al., 2023)
Wheat	RGB, HS-NIR	SR, Th, Tx	ELM	R^2^ = 0.74	China	(Ma et al., 2023)
Wheat	MS	VIs	RR	R^2^ = 0.651	Spain	(Gracia-Romero et al., 2023)
Rice	HSI	ID	LR	R^2^ = 0.858 and RMSPE = 7.52%	Japan	(Kurihara et al., 2023)
Wheat	SR	ND-RE	PLSR	R^2^ = 0.81	Germany	(Prey et al., 2023)
Rice	MS	VIs, TIs	RF	R^2^ = 0.795, RMSE = 0.298, RRMSE = 0.072	China	(Longfei et al., 2023)
Maize	RGB, MS	ID	RF	R^2^ = 0.859, RMSE = 1086.412 kg/ha, RMSE = 13.1%	China	(Liu et al., 2023)
Maize	MS, SR	VIs	ANN, DT, REPT, RF, SVM	R = 0.58	Brazil	(Baio et al., 2022)
Wheat	MS	CIs	PLSR	R^2^ = 0.81, RMSE = 1248.48, NRMSE = 21.77%,	China	(Wang et al., 2022a)
Wheat	RGB, MS	VIs	SVM, RF, PLSR, RR, MLR	R^2^ = 0.85	Germany	(Prey et al., 2022)
Rice	MS	VIs, TIs	RF	RMSE = 0.94 t/ha, RRMSE = 9.37%	China	(Zheng et al., 2022)
Maize	MS	VIs	PCA, LR, R	R^2^ = 0.61	Peru	(Saravia et al., 2022)
Wheat	MS	VIs	MLR, SMLR, PLSR	R^2^ = 0.61, RMSE = 7.48 kg yield/kg N, MAE = 6.05 kg yield/kg N	China	(Liu et al., 2022)
Rice	SAR images	Ku band	WCM	92.7%	China	(Wang et al., 2022b)
Wheat	MS	VIs	RF	R^2^ = 0.8516, RMSE = 0.0744 kg/m2	China	(Tian et al., 2022)
Wheat	SR, HSI	VIs	PLSR, ANN	RMSE = 599.63 kg/ha, NRMSE = 9.82%	China	(Feng et al., 2022)
Rice	MS	VIs	TCT	83%	China	(Luo et al., 2022)
Wheat	RGB, TIR, MS	VIs	SVM, DNN, RR, RF, EL	R^2^ = 0.692	China	(Fei et al., 2023)
Rice	MS	VIs	XGB	R^2^ = 0.83	Japan	(Bascon et al., 2022)
Maize	RGB, MS, SR	DF	RF, DCN, RF, DCN, SVM	RRMSE = 17.22%	China	(Yu et al., 2023)
Wheat	TIR, MS	VIs, WIs	NN, RF	R^2^ = 0.78, RRMSE = 684.1 kg/ha	China	(Shen et al., 2022)
Wheat	OP	SEL	YOLOX-m	87.93%	China	(Zhaosheng et al., 2022)
Wheat	HSI	FFLR	PLSR	86.58%	USA	(Costa et al., 2022)
Maize	MS	SIs, TIs	RF	R^2^ = 0.93	China	(Yang et al., 2022)
Maize	MS	3D-CNN, 2D-CNN	XGB	RMSE: 8.7–9.3%	USA	(Bellis et al., 2022)
Maize	MS, DI	SIs, TIs	TCM	R² = 0.82, RMSE = 38.53 g/m², RRMSE = 29.19%	China	(Meiyan et al., 2022)
Wheat	MS	VIs	GPR, SVR, RFR	R^2^ = 0.88, RMSE = 49.18 g/m^2^	China	(Bian et al., 2022)
Soybean	MS	VIs	gSW, BMT	R^2^ = 0.98	Brazil	(Tavares et al., 2022)
Soybean	MS	OMI	LASSO, PCA,	76%	USA	(Zhou et al., 2022)
Wheat	HSI	SIs, FS	SVM, GP, LRR, RF	R^2^ = 0.78	China	(Li et al., 2022)
Wheat	T, MS	VIs, DF, NRCT	ENR, EWF	R^2^ = 0.729, RMSE = 0.831 t/ha	China	(Fei et al., 2021b)
Rice	RGB	VIs	RF	R^2^ = 0.80	China	(Ge et al., 2021)
Soybean	MS	SBs, VIs	RF, SVM, LR	RMSE = 8.23, MAE = 6.65	Brazil	(Teodoro et al., 2021)
Maize	MS	VIs	DNN	RMSE = 1.07 t/ha, R^2^ = 0.73, RRMSE = 7.60% t/ha	Australia	(Danilevicz et al., 2021)
Maize	MS	VIs	EM	R^2^ = 0.97	USA	(Sunoj et al., 2021)
Rice	HSI	SIs, TIs	MLR	R^2^ = 0.80, RMSE = 0.421 Mg/ha	China	(Wang et al., 2021b)
Wheat	MS, 3D point cloud, SR,	VIs	MTLR, SVM, GPR, ANN	R^2^ = 0.88; RMSE = 11.8 g/m^2^	Australia	(Roy Choudhury et al., 2021)
Rice	MS	VIs	MLR	R^2^ = 0.95	Greece	(Perros et al., 2021)
Wheat	MS	WIs, VIs	LRM	R^2^ = 0.87	Canada	(Song et al., 2021)
Rice	MS	VIs, FIs	MLR	R^2^ = 0.869, RMSE = 396.02 kg/ha, MAPE= 3.98%	China	(Wang et al., 2021a)
Wheat	RGB	CIs	SVM	RMSE = 32.18 g/m^2^, R^2^ = 0.93	China	(Zeng et al., 2021)
Wheat	MS	VIs	RF, SVM, GP, RR	R^2^ = 0.628	China	(Fei et al., 2021a)
Rice	MS	VIs	SMA, BMM	91.9%	China	(Yuan et al., 2021)
Wheat	TI	TeIs, WIs	CRT	RMSE = 16.7 g/m^2^, R^2^ = 0.78,	Australia	(Das et al., 2021)
Wheat	MS	VIs	MLR	R^2^ = 0.807, RMSE = 781.59 kg/ha	China	(Han et al., 2021)
Rice	MS	VIs	NN	92.9%	China	(Duan et al., 2021)
Wheat	MS	GAI	RA	R^2^ = 0.82	Germany	(Bukowiecki et al., 2021)
Maize	MS	VIs	MLR, DT	R = 0.86, RMSE = 0.32	South Africa	(Chivasa et al., 2021)
Maize	HSI	TI, SI	CNN	75.50%	China	(Yang et al., 2021b)
Wheat	MSI	VIs	SFS, LASSO-R, SVR	90%	Norway	(Shafiee et al., 2021)
Wheat	RGB, MS	VIs	DSF, OMI	R^2^ = 0.70	USA	(Bhandari et al., 2021)
Maize	MS	VIs	EKF	R^2^ = 0.855, RMSE = 692.8kg/ha	China	(Peng et al., 2021)
Soybean	MS	VIs	CNN, DNN, RNN	RMSE = 391 kg/ha	USA	(Zhou et al., 2021a)
Wheat	DI, HSI	VIs, TIs	PLSR, SVM	R^2^ = 0.87, RMSE = 119.76 g/m^2^	China	(Fu et al., 2021)
Wheat	HSI	VIs	RF	RMSE = 985.83 (kg/ha)	China	(Yang et al., 2021a)
Maize	MS	VIs	LMvR	R^2^ = 0.62	Nigeria	(Adewopo et al., 2020)
Soybean	HSI	VIs, DN	PLSR	R^2^ = 0.79,	China	(Li et al., 2021)
Maize	MS	VIs	RF	R = 0.78, MAE = 853.11kg/ha	Brazil	(Ramos et al., 2020)
Wheat	RGB	VIs	PSO	R^2^ = 0.63, RMSE = 1.16 t/ha, MAE = 0.96 t/ha, NRMSE = 21.9%	China	(Yue et al., 2021)
Maize	RGB	3D-PC, OMI	LR	R^2^ = 0.94	France	(Gilliot et al., 2021)
Rice	RGB, MS	VIs, SI	RF	R^2^ = 0.85, RRMSE = 3.56%	China	(Wan et al., 2020)
Wheat	MS, SR	VIs	LR, RF, ANN	RMSE = 1.07%	Japan	(Zhou et al., 2021b)
Maize	RGB	VIs	BP, ExLM, SVM, RF	MAEs = 0.925 g/hundred grain weight	China	(Guo et al., 2020)
Wheat	RGB	DSF	LEER	R^2^ = 0.73	Nepal	(Panday et al., 2020)
Wheat	RGB	LAI	SCE	RRMSE = 15.2%	Canada	(Song et al., 2020)
Maize	RGB, MS	VIs	LM, GA	R = 0.97, RMSE = 0.425 t/ha, MAE = 0.249 t/ha	Mexico	(García-Martínez et al., 2020)
Soybean	RGB, MS	PC	RF, XGB	91.36%	USA	(Herrero-Huerta et al., 2020)
Wheat	MS	VIs	LR, MLR, SMLR, PLSR, ANN, RF	R^2^ = 0.78, RRMSE = 0.1030.	China	(Fu et al., 2020)
Maize	HSI	VIs	PLSR	RMSE = 2.07 ton/ha, R^2^ = 0.73,	Israel	(Herrmann et al., 2020)
Soybean	HSI, Th, Tx	VIs	PLSR, RFR, SVR, DNN	R^2^ = 0.720, RMSE= 15.9%	USA	(Maimaitijiang et al., 2020)
Wheat	HSI	SIs	PLSR, ANN, RF	R^2^ = 0.77, NRMSE = 10.63%, RMSE = 648.90 kg/ha	China	(Tao et al., 2020)
Wheat	RGB	VIs	PCA,	R^2^ = 0.67	Italy	(Marino and Alvino, 2020)
Maize	RGB, MS	VIs	NRM	85-94%	China	(Zhang et al., 2020)

### 4.1 Utilization of machine learning techniques and factors influencing yield prediction

Various methodologies and techniques have been employed in previous findings for YP. **Table 4** provides details of the ML approaches applied in recent studies. The RF technique is widely employed in the domain of YP in WMRS crops, as it is a commonly utilized method in technical approaches. The studies have used numerous techniques, i.e. SVM, KNN, PCA, LR, MLR, MMDL, RR, ANN, PLSR DCN, etc. SVM can be used with statistical techniques like ANOVA to estimate agricultural yield (Li et al., 2018). In wheat crop SVM manifested R^2^ = 0.93 using RGB images acquired by UAVs (Wang et al., 2021a). Another study established the R^2^ = 0.87 for wheat crops through MS images by UAVs calculating WIs and VIs using the LRM technique (Song et al., 2021). The MLR showed R^2^ = 0.81 for wheat crop (Han et al., 2021) and R^2^ = 0.95 for rice crop through MS images (Perros et al., 2021). Accordingly, different ML techniques have shown interesting results using raw image data acquired using UAVs (**Table 4**). The details of the abbreviations is given in **Table 5**.

**Table 5:** List of abbreviations for acronyms used in the manuscript.

Acronyms	Abbreviations	Acronyms	Abbreviations
3D-PD	3D-point cloud	MSMA	Member spectral mixture analysis
A	Abundance	MTLR	Multitarget linear regression
ANN	Artificial Neural Network	ND-RE	Normalized difference red edge
BMM	Bilinear mixing model	NN	Neural network
BMT	Box-M test	NRCT	Normalized relative canopy temperature
BP	Backpropagation neural network model	NRM	Nonlinear regression models
CIs	Color indices	NRMSE	Normalized root-mean-square error
CNN	Convolutional neural network	OMI	Orthomosaic images
CRT	Classification and regression tree	OP	Orthophotos
DCN	Deep convolutional network	PC	Point clouds
DF	Data fusion	PCA	Principal component analysis
DI	Digital images	PLS	Partial least squares
DN	Digital numbers	PLSR	Partial least squares regression
DNN	Deep Neural Network	PSO	Particle swarm optimization,
DSF	Digital surface model	R	Pearson correlation coefficient
DT	Decision Tree	R^2^	Co-efficient of determination
EKF	Ensemble Kalman Filter	RA	Regression analysis
ELM	Ensemble learning model	RE	Red-edge
EM	Exponential models	REPT	REPTree Decision Tree
ENR	Elastic net regression	RF	Random forest
EWF	Entropy weight fusion	RFR	Random forest regression
ExLM	Extreme learning machine	RMSE	Root mean square error
FFLR	Function on function linear regression	RMSPE	Root mean square percentage error
FIs	Fluorescence Indices	RNN	Recurrent neural network
FS	Feature selection	RR	Ridge regression
GA	Garson’s Algorithm	RRMSE	Relative root mean square error
GAI	Green area index	SA	Sun angles
GB	Gradient boost	SBs	Spectral bands
GF	Gap fraction	SCE	Shuffled Complex Evolution
GL	Generalized linear	SI	Spectral information
GP	Gaussian process	SEL	Squeeze-and-Excitation Layer
GPR	Gaussian process regression	SFS	Sequential forward selection
gSW	Generalized Shapiro–Wilk	SIs	Spectral Indices
HSI	Hyperspectral imaging	SMLR	Stepwise MLR
HS-NIR	Hyperspectral Near infrared	SR	Spectral reflectance
ID	Index development	SVM	Support vector machine
KNN	k-Nearest Neighbor	SVR	Support vector regression
LAI	Leaf area Index	TCM	Tridimensional concept mode
LASSO-R	Least absolute shrinkage and selection operator regression	TCT	Tasseled cap transformation
LD	Layer depths	TeIs	Temperature Indices
LEER	Linear, exponential and empirical regression	Th	Thermal
LM	Levenberg–Marquart	TI	Thermal imaging
LMvR	Linear multivariate regression	TxI	Texture Information
LR	Linear Regression	TIR	Thermal infrared
LRM	Linear regression model	TIs	Texture Indices
LRR	Linear ridge regression	Tx	Texture
MAE	Mean absolute Error	VIs	Vegetation Indices
MAPE	Mean absolute percentage error	WCM	water-cloud model
MLR	Multiple linear regression	WD	Weather data
MMDL	Multimodal deep learning	WIs	Water indices
MS	Multispectral	XGBoost	Extreme gradient boost

The authors give similar importance to each factor for inclusion into the ML model (Zhang et al., 2019; Mustafa et al., 2022b). Osco et al. (2020) consider the significance of several elements like meteorological conditions, geographic location, and radiometric calibration. Each location’s meteorological variables (daily daylight hours, daily solar radiation, daily temperature sum, and daily wind speed) were employed, along with field-specific rainfall data. They found that the number of neural layers and the learning rate of an ANN affect the prediction. As a result, the prediction model performs worse when too many or too few parameters are added (Adisa et al., 2019). The conclusion highlights the significance of neural networks in creating prediction models. On the other hand, by including irrelevant features to the model, its complexity may increase, leading to a drop in prediction accuracy concerning time complexity and irrelevant results (Kanning et al., 2018). The study Eugenio et al. (2020) indicates that the UAV is the most effective method for gathering image data from the location. They performed their experiment accounting for distinct weather scenarios. Overall performance shows the accuracy of the model, although this study has indicated that the inclusion of either an excessive number or a small number of factors causes data sparsity. The main problem this study highlighted is that they employ constant weather conditions for every season. Because of this, a realistic strategy is required, considering the various weather-related influencing factors throughout the year. Peng et al. (2019) has mentioned that ML technology is typically used in precision agriculture to leverage the vast amounts of collected data. ML can estimate certain crop growth rate-related metrics, identify and differentiate objects in photos, and even detect diseases. To take advantage of the data, ML techniques such as CNN, ANN, regression modeling, RF, and deep learning have been employed (Islam et al., 2018). The use of ML has grown dramatically in recent years, partly because deep learning is developing at a rapid pace (Guo et al., 2019; Mustafa et al., 2022a, 2022; Hussain et al., 2023; Mustafa et al., 2024).

### 4.2 Emerging trends and the current research landscape in the domain of yield prediction in WMRS crops utilizing UAVs

Some of this study’s most substantial implications are as follows:

- Regarding YP in WMRS crops utilizing UAVs research, Zhou X, Maimaitijiang M, Yue JB, Jin XL, Bendig J, Haboudane D, and other writers are the most productive at the micro level. Researchers like Shanahan et al. (2001); Chlingaryan et al. (2018); Maes and Steppe (2019); Zheng et al. (2019), and others have been referenced extensively in YP for WMRS crops utilizing UAVs.
- Beijing Academy of Agriculture Forestry Science, Ministry of Agriculture Rural Affairs, Chinese Academy of Agricultural Sciences and China Agriculture University are the most dynamic and productive research for YP in WMRS crops utilizing UAVs at the meso scale.
- China, the United States, Germany, Australia, Canada, Spain, and Brazil are the most active and productive contributors to YP in WMRS crops using UAVs at the macro level. Compared to the other countries on the list, China and the United States presumably have more publications because their governments provide more extensive financial support policies to the scientific community.
- The National Natural Science Fund of China, the National Key Research and Development Program of China, the National Science Foundation, the Fundamental Research Funds for The Central Universities, and others have provided the bulk of the funding for YP in WMRS crops using UAVs.
- Precision Agriculture, Computers and Electronics in Agriculture, Remote Sensing, and Frontiers in Plant Science supported the main journals most.
- Although promising progress has been made in predicting crop yields with WMRS employing UAVs, more definitive results are urgently needed.

These findings provide important insight into the state of research and development in YP in WMRS crops using UAVs, and they highlight the most recent advancements in the field.

## 5 Constraints and prospects for the future

Despite the extensive research performed on YP of WMRS crops using RS, a definitive technique or technological configuration can still be universally applied. Due to variations in ML approaches, the selected or retrieved specific features such as vegetation indices, color, and spectral properties exhibit fluctuations (Zheng et al., 2019; Baio et al., 2022). **Table 4** displays the suitability and chaos of several algorithms. As a result, the primary future vision is to concentrate on certain methodology that can be generalized for accurate quantification and YP.

Examining complex interactions among many environmental conditions will enable more precise and real-time crop YPs (Jung et al., 2021; Mia et al., 2023). A more thorough dataset for enhancing prediction models can be produced by establishing data standards protocols and encouraging data exchange between farmers and researchers; collaboration can result in more accurate insights and forecasts (Wolfert et al., 2017). Additionally, efforts to make UAVs technology less expensive and sophisticated will enhance its use, which will be advantageous to small-scale farmers. While incentives and subsidies provided by the government may be helpful. Crop YP and agriculture management benefit from deep and ML. These new algorithms can more effectively analyze complicated environmental interactions and massive datasets than older methods, resulting in more accurate and real-time yield estimates. Deep learning algorithms can identify complex data patterns for better predictions and insights (Maimaitijiang et al., 2020; Bellis et al., 2022; Mia et al., 2023; Yu et al., 2023).

Additionally, improvements in sensor technology (hyperspectral and LiDAR sensors) can offer even more precise and detailed information, enabling crop health and yield potential (Maes and Steppe, 2019). Also, it will be necessary to push for simplified laws that balance safety issues and the potential advantages of UAVs technology in agriculture. This may encourage a broader uptake of UAVs (McCarthy et al., 2023). Most significantly, maximizing the effectiveness of UAVs solutions for certain crops and areas, customized sensors and data analysis methods can give farmers and researchers more pertinent information.

## 6 Contribution of the study for the scientific community

An in-depth scientometric analysis of academic literature from 2001 to 2023 highlights research trends, boundaries and institutional relationships. The study identifies critical gaps, such as reliance on English-language articles and the need for transferable culture-specific features between sensors and algorithms. It proposes standardized data protocols for consistency and comparability, advanced hybrid models combining multiple machine learning (ML) algorithms and remote sensing (RS) techniques, and real-time monitoring systems using machine learning and deep learning to gain immediate insights. In addition, it recommends integrating advanced sensors such as hyperspectral and LiDAR with UAVs, promoting collaboration and data sharing between stakeholders, and advocating balanced government incentives and regulations for UAVs. These contributions aim to mitigate current limitations, ensure more accurate, reliable and widely applicable yield forecasts, and ultimately improve global agricultural productivity.

## 7 Conclusion

This study conducted a scientometric analysis of the academic literature pertaining to the use of unmanned aerial vehicles (UAVs) for yield prediction (YP) in Wheat, Maize, Rice, and Soybean (WMRS) crops. The retrieval of research published between 2001 and 2023 was conducted using co-citation, co-authorship, and co-occurrence analysis of phrases, utilizing the Web of Science (WOS) database. Research frontiers, trending issues, authors, innovative knowledge systems, and institutional relationships were all taken into account. Although the results of the present study’s graphical analysis of related papers are outstanding, the study has many shortcomings. Since the databases that make up the WOS core collection only include English-language articles, the resulting reference footprint is limited. Although remote sensing can predict yields with high accuracy, more research is needed, focusing on identifying transferable crop-specific traits across sensors and algorithms. This would make it possible to estimate yields more precisely and consistently, enhancing crop management techniques, reducing financial losses, and guarantee food security. Thus, research must concentrate on extracting quantitative knowledge on various crop stages utilizing various data types to construct comprehensive decision support systems. These advancements in remote sensing and YP have the potential to increase agricultural productivity across the globe significantly. Moreover, application of the advanced hybrid models, data protocols and feature selection are appealing indicators in conjunction with ML and RS for WMRS crops’ yield estimation. Government incentives for farmers, real time monitoring using ML and deep learning, hyperspectral and Lidar sensors, and UAV regulations can enhance the YP investigations.

## Data Availability

The raw data supporting the conclusions of this article will be made available by the authors, without undue reservation.
