# Supplementary material for: Establishing a knowledge structure for yield prediction in cereal crops using unmanned aerial vehicles
Source: Front Plant Sci. 2024 Aug 9;15:1401246. doi: 10.3389/fpls.2024.1401246 (PMC11341481; doi:10.3389/fpls.2024.1401246)
Supplement: Supplementary file 1 [file Table_1.docx]

Supplementary Information

Establishing a Knowledge Structure for Yield Prediction in Cereal Crops Using Unmanned Aerial Vehicles

Ghulam Mustafa^1,2^, Imran Haider Khan^2^, Sarfraz Hussain^3^, Yuhan Jiang^1^, Jiayuan Liu^1^, Saeed Arshad^2^, Raheel Osman^4^, Yuhong Liu^1*^

^1^ Key laboratory of integrated regulation and resource development on shallow lakes, ministry of education, College of environment, Hohai university, Nanjing 210098, China.

^2^ College of Agriculture, Nanjing Agricultural University, Nanjing 210095, China.

^3^ College of Physics and Optoelectronic Engineering, Shenzhen University, Shenzhen 518060, China.

^4^ Department of Agronomy, Iowa State University, United States of America.*** Correspondence:**Yuhong Liu
yhliu@hhu.edu.cn

| Table S1 The 15 best countries for publishing research on yield prediction in WMRS crops utilizing UAVs | | | |
| --- | --- | --- | --- |
| No. | Countries | Records | % of total |
| 1 | PEOPLES R CHINA | 96 | 42.478 |
| 2 | USA | 45 | 19.912 |
| 3 | GERMANY | 18 | 7.965 |
| 4 | AUSTRALIA | 16 | 7.080 |
| 5 | CANADA | 11 | 4.867 |
| 6 | SPAIN | 11 | 4.867 |
| 7 | BRAZIL | 8 | 3.540 |
| 8 | JAPAN | 8 | 3.540 |
| 9 | INDIA | 6 | 2.655 |
| 10 | ITALY | 6 | 2.655 |
| 11 | EGYPT | 5 | 2.212 |
| 12 | ENGLAND | 5 | 2.212 |
| 13 | SAUDI ARABIA | 4 | 1.770 |
| 14 | BELGIUM | 3 | 1.327 |
| 15 | FINLAND | 3 | 1.327 |
| Table S2 The 15 best articles for research on yield prediction in WMRS crops utilizing UAVs | | | |
| Sr. No. | Cited References | Count | Year |
| 1 | (Zhou et al., 2017) | 48 | 2017 |
| 2 | (Maimaitijiang et al., 2020) | 32 | 2020 |
| 3 | (Jiang et al., 2008) | 28 | 2008 |
| 4 | (Bendig et al., 2014) | 28 | 2014 |
| 5 | (Wang et al., 2014) | 24 | 2014 |
| 6 | (Geipel et al., 2014) | 24 | 2014 |
| 7 | (Bendig et al., 2015) | 24 | 2015 |
| 8 | (Yang et al., 2019) | 23 | 2019 |
| 9 | (Hassan et al., 2019) | 23 | 2019 |
| 10 | (Yue et al., 2017) | 22 | 2017 |
| 11 | (Yue et al., 2019) | 21 | 2019 |
| 12 | (Wan et al., 2020) | 21 | 2020 |
| 13 | (Yang et al., 2017) | 20 | 2017 |
| 14 | (Maimaitijiang et al., 2017) | 20 | 2017 |
| 15 | (Fu et al., 2014) | 20 | 2014 |
| Table S3 The 15 best funding agencies for publishing research on yield prediction in WMRS crops utilizing UAVs | | | |
| No. | Funding agencies | Records | % of total |
| 1 | National Natural Science Foundation of China NSFC | 46 | 20.354 |
| 2 | National Key Research and Development Program of China | 13 | 5.752 |
| 3 | National Science Foundation NSF |  |  |
| 4 | Fundamental Research Funds for The Central Universities | 8 | 3.540 |
| 5 | Central Public Interest Scientific Institution Basal Research Fund | 7 | 3.097 |
| 6 | National High Technology Research and Development Program of China | 5 | 2.212 |
| 7 | National Key R D Program of China | 5 | 2.212 |
| 8 | Spanish Government | 5 | 2.212 |
| 9 | Beijing Natural Science Foundation | 5 | 2.212 |
| 10 | Conselho Nacional De Desenvolvimento Cientifico E Tecnologico Cnpq | 4 | 1.770 |
| 11 | Coordenacao De Aperfeicoamento De Pessoal De Nivel Superior Capes | 4 | 1.770 |
| 12 | Federal Ministry of Education Research Bmbf | 4 | 1.770 |
| 13 | Grants In Aid for Scientific Research Kakenhi | 4 | 1.770 |
| 14 | Japan Society for The Promotion of Science | 4 | 1.770 |
| 15 | Ministry Of Education China 111 Project | 4 | 1.770 |
